# Dissecting the HGT network of carbon metabolic genes in soil-borne microbiota

**DOI:** 10.3389/fmicb.2023.1173748

**Published:** 2023-07-07

**Authors:** Liangzhi Li, Yongjun Liu, Qinzhi Xiao, Zhipeng Xiao, Delong Meng, Zhaoyue Yang, Wenqiao Deng, Huaqun Yin, Zhenghua Liu

**Affiliations:** ^1^School of Minerals Processing and Bioengineering, Central South University, Changsha, China; ^2^Key Laboratory of Biometallurgy of Ministry of Education, Central South University, Changsha, China; ^3^Hunan Tobacco Science Institute, Changsha, China; ^4^Yongzhou Tobacco Company of Hunan Province, Yongzhou, China; ^5^Hengyang Tobacco Company of Hunan Province, Hengyang, China; ^6^Changsha Institute of Agricultural Science, Changsha, China

**Keywords:** soil, microbiota, horizontal gene transfer, network, carbon metabolic gene

## Abstract

The microbiota inhabiting soil plays a significant role in essential life-supporting element cycles. Here, we investigated the occurrence of horizontal gene transfer (HGT) and established the HGT network of carbon metabolic genes in 764 soil-borne microbiota genomes. Our study sheds light on the crucial role of HGT components in microbiological diversification that could have far-reaching implications in understanding how these microbial communities adapt to changing environments, ultimately impacting agricultural practices. In the overall HGT network of carbon metabolic genes in soil-borne microbiota, a total of 6,770 nodes and 3,812 edges are present. Among these nodes, phyla Proteobacteria, Actinobacteriota, Bacteroidota, and Firmicutes are predominant. Regarding specific classes, Actinobacteria, Gammaproteobacteria, Alphaproteobacteria, Bacteroidia, Actinomycetia, Betaproteobacteria, and Clostridia are dominant. The Kyoto Encyclopedia of Genes and Genomes (KEGG) functional assignments of glycosyltransferase (18.5%), glycolysis/gluconeogenesis (8.8%), carbohydrate-related transporter (7.9%), fatty acid biosynthesis (6.5%), benzoate degradation (3.1%) and butanoate metabolism (3.0%) are primarily identified. Glycosyltransferase involved in cell wall biosynthesis, glycosylation, and primary/secondary metabolism (with 363 HGT entries), ranks first overwhelmingly in the list of most frequently identified carbon metabolic HGT enzymes, followed by pimeloyl-ACP methyl ester carboxylesterase, alcohol dehydrogenase, and 3-oxoacyl-ACP reductase. Such HGT events mainly occur in the peripheral functions of the carbon metabolic pathway instead of the core section. The inter-microbe HGT genetic traits in soil-borne microbiota genetic sequences that we recognized, as well as their involvement in the metabolism and regulation processes of carbon organic, suggest a pervasive and substantial effect of HGT on the evolution of microbes.

## 1. Introduction

Microorganisms are the foundation of the Earth’s biosphere and play an integral and unique role in various ecological processes and functions, where they interact to form complex functional networks (Zhou et al., 2010). Soil is an important component of the global carbon cycle and is critical to climate change mitigation. Almost all life on Earth cannot leave the living soils, which emphasizes the significance of soil-borne microbiota for essential life-supporting processes (e.g., carbon and nitrogen cycling). The soil organic carbon (SOC) reservoir (about 1,500 Gt) is supposed to be greater than the sum of the carbon stocks in the air and global flora (about 560 Gt) (van Elsas, 2019; Jassey et al., 2022). Microorganisms have much higher growth rates and carbon turnover rates than plants. The size of the soil organic carbon pool depends to a large extent on microorganisms, as their growth and activity balance the accumulation of organic carbon and its release through the decomposition of plant die-offs. Soil-borne microbes control the kinetics of soil carbon transformation by converting carbon from plants, incorporating carbon resources to increase biomass, and breaking down terrestrial organic compounds. For instance, strong biological methane-oxidizing activities in agricultural soils can lead to the emissions of biogenic CO_2_ linked to CH_4_ oxidation by a large biodiversity of methanotrophs (Cappelletti et al., 2016). Globally, soil algae absorb about 3.6 Pg of carbon per year, which is 6.4% of annual terrestrial net primary productivity (NPP) and equivalent to 31% of global anthropogenic carbon emissions (Jassey et al., 2022). Soil-borne microflora is not only dynamic temporally but also varied geographically. As a result, fluctuations in the activities and quantity of the communities that constitute such soil-borne microflora are frequently seen (Fierer, 2017). The heterogeneity in soil-borne microbiota composition is primarily caused by the spatial heterogeneity of amounts and concentrations of, for example, nutrition, mineral resources, pH, and moisture in the soil mass (Fierer, 2017; van Elsas, 2019), as well as shaped by genetic recombination and gene-specific selection processes (Crits-Christoph et al., 2020). Besides, the carbon resource abundance and diversity in soil have been proven to correlate the ecological certainty during the bio-control of microbe-induced plant disease (Zhou et al., 2023).

Horizontal gene transfer (HGT), or the interchange of genetic material across phylogenetic clades, is thought to be an efficient strategy for dispersing reproductive fitness for prokaryotic and eukaryotic cells (Huang, 2013; Daubin and Szöllősi, 2016; Li et al., 2022). Employing transmitting mobile genetic elements (MGEs) like plasmids, viruses, transposons, and gene transfer agents (GTA, tailed phage-like entities capable of packing and transferring random pieces of the host genome) as well as by direct absorption and assimilation of naked DNA by homologous or unauthorized recombination, new genes are transferred through HGT (Thomas and Nielsen, 2005; Lang and Beatty, 2007). As a result, the origins of specific genes within a particular species can vary, and patterns of gene exchange between near and remotely affiliated lineages can be seen on various genomes (Gogarten et al., 2002; Koonin, 2005; Kunin et al., 2005). HGT is commonly described based on contradicting phylogenetic trees upon genomic comparison (Delsuc et al., 2005; Li et al., 2019). In recent decades, the evolution and HGT processes of genetic traits have been investigated in a variety of ecosystems, where multiple factors (e.g., environmental conditions, ancestral genome sizes) might have influenced the frequency of HGT during evolution (Huang, 2013; Daubin and Szöllősi, 2016; Li et al., 2022). Stable HGT flux induced under selection was also suggested to enhance microbial interplay’s structural stability and thereby maintain microbial communities’ equilibrium (Fan et al., 2018). For instance, it was reported that 9.6% of the genes within a prokaryotic genome were recently acquired on average (Kloesges et al., 2011), while in *E. coli* 18% of genes were recently acquired *via* HGT (Lawrence and Ochman, 2002), and in Rickettsiales 25% of core genes were recently transferred (Hernández-López et al., 2013).

Even though the terrestrial area is a continually fluctuating and difficult place (Fierer, 2017), factors like limited nutritional and ion intensities that endorse competency, clay deposits that sustain the perseverance of bacteriophages and naked DNA, and the capacity of subsoil microbes to aggregate for genetic exchange suggest that HGT processes could be performing in this globalized context. Consistently, the transmission of antibiotic-resistance genes in the soil *via* MGEs (e.g., bacteriophage) has been highlighted as a public concern (Gallo et al., 2019; Zhang et al., 2022). Besides, soil is stratified geographically and varied physiologically and biologically on various dimensions, which offers a unique habitat for microbiota of diverse background. Many agronomic factors, such as pH, saltiness, warmth, and humidity, influence the organization of the microbiota inside the complicated soil-borne biomass (Frindte et al., 2019). The microscopic society’s shape and function in soils could also be influenced by temporal changes such as meteorological conditions, rhizosphere exudation, as well as other periodical supplies of plant organic matter (Zheng et al., 2019; Chernov and Zhelezova, 2020). In natural soil settings, the first investigative research on HGT among microorganisms was conducted in the 1970s (Weinberg and Stotzky, 1972; Graham and Istock, 1978). Since then, further investigations have employed fieldwork and microcosm experiments to evaluate ecological HGT (van Elsas et al., 2006). Plant-associated soils and biofilm communities are well-known hotspots for HGT due to the great genetic variety on such a restricted geographical level (Fan et al., 2018). It was found that the genetic factors in HGT are more abundant in the rhizosphere than in bulk soil, therefore HGT may aid in the development of rhizosphere competence (Vieira et al., 2020). Previous bipartite network analyses have provided evidence of genetic exchange, plasmid fusion and fission, exogenetic plasmid transfer, and environmental adaptation of the soil bacteria such as *Rhizobium* (Corel et al., 2018; Li L. et al., 2020; Li X. et al., 2020). In addition, plasmid transmission from transplanted *P. fluorescens* to native gram-negative rhizobacteria in soil has been proven to happen in natural settings (Balthazar et al., 2021).

Many terrestrial subterranean microbes have also been found to contain MGEs, and some of them have already shown conjugative activity in a lab environment or even in bulk soil. Solitary (Feng et al., 2007) or multiple (Brockman et al., 1989) plasmids could be present in subterranean isolates, with big plasmids that are more apt to own recombinant functionality seeming to predominate (Fredrickson et al., 1988). Microorganisms buried deeper underground have been shown to harbor extremely large (>150 kb) conjugative plasmids at a higher rate than microorganisms from shallower subsurface soils (Ogunseitan et al., 1987; Fredrickson et al., 1988). Degradative genetic determinants are commonly found in subsurface large plasmids, which significantly increase the host’s metabolism adaptability (Romine et al., 1999; Basta et al., 2004). Elements similar to recombinases are also present in several accessible plasmids, implying the possibility of incorporation and excision from the soil microbial genome (Romine et al., 1999). Among the sequenced soil subsoil genomes, *G. thermodenitrificans* was discovered to match 11 and 3% of its genetic makeup with *Bacillus* sp. and other Firmicutes, respectively. Around 2.7% of the genes in *G. thermodenitrificans* NG80-2 exclusively occur in distant relatives and may have undergone HGT. This includes two groups of proteins associated with nitrogen use that seem to have originated separately. The addition of such genes and the catabolic plasmid pLW1071 has significantly improved the metabolic adaptability in nutrient-poor conditions (Feng et al., 2007). Furthermore, the introduction of MGEs into synthesized microbial communities (SynComs) was considered an effective way for manipulating the microbial community for applicable purposes (e.g., carbon storage) (Song et al., 2014; Tsoi et al., 2019; de Lorenzo, 2022).

Yet, unlike simple bacterial mixes in a lab, the complexities of HGT mechanisms in wild areas, including the heavily heterogeneous populations, still need to be fully deciphered. *In situ* critical cellular events, additional chemical triggers for genetic exchange or absorption, and natural factors like cell-MGE interactions are examples of the complexities. In addition, there needs to be more focus on the environmental implications, such as how these HGT activities affect microbial communities’ capacity to adapt to wild areas, and how these HGT mechanisms reflect the features of the particular ecosystem. Though there is a growing interest in elucidating the underlying microbial mechanisms driving soil carbon transformation, stabilization, and release processes, many unknowns remain. Nonetheless, many earlier studies mainly concentrated on HGT episodes of particular lineages and on significant issues such as adaption-associated processes (Li et al., 2019; Li L. et al., 2020; Li X. et al., 2020; Li et al., 2021). Regardless of these efforts, there is so much to be learned in a holistic mode about the quantity and effects of HGT-driven genetic makeup in soil-borne microbiota that can confer a variety of carbon assimilation and dissimilation functions (e.g., central carbon cycles, fatty acid biosynthesis). This poses significant challenges to understanding gene flow in terrestrial microbial communities. As a result, it remains to be seen whether there are inter-microbe HGT(s) that have promoted the development of soil-borne microbiota in a variety of conditions. If these HGT markers exist, how do they work to counteract environmental stressors, and how widely disseminated are these markers throughout the genetic material of soil-borne microbiota?

A variety of natural systems, including protein expression (Alon, 2007), biochemical processes (Pál et al., 2005), biomolecule interplay (Jeong et al., 2001), contradicting evolutionary indications (Huson and Bryant, 2006), and ecosystem dynamics (Rezende et al., 2007), are being modeled using network infrastructure (Proulx et al., 2005). In theory, the network is capable of better displaying the patterns of microbial genomic evolution (Doolittle and Bapteste, 2007). HGT networks are a distinctive form of sharing gene networks. They are intended to investigate trends in genetic dispersion brought on by HGT throughout ecological evolution.

Under this context, we performed BLASTP-driven screenings and HGT network constructions to discover and investigate potential inter-microbe HGT genes linked to carbon metabolism throughout genomic sequences of all accessible strains isolated from soils, followed by network constructions. Our results suggested that the HGT episodes may significantly contribute to genetic imports that contribute to the soil-borne microbiota’s increased carbon metabolic versatility and adaptivity. Our knowledge of microbial interplay and the adaptable development of microbes to deal with various situations have therefore been enhanced as a result of this work. However, further experimental work is still necessary to evaluate the occurrence of HGT in the vast and unexplored environment of the terrestrial subsurface.

## 2. Results

### 2.1. Overall statues

We first queried and retrieved all the items in the public database (Genbank/IMG) with the keyword “soil” within the “biosample” or “habitat” regions. We then chose and downloaded from the public database (Genbank/IMG) the out-coming high-quality genomes of soil microbial isolates (*n* = 764, see Supplementary Table S1 for details at https://doi.org/10.6084/m9.figshare.22154828.v1) for downstream analyses. Putative microbial horizontally transferred genes (HTGs) in these genomes were identified employing the BLASTP-based IMGAP pipeline under default mode (Markowitz et al., 2010), which resulted in a total of 37,481 HTG entries, followed by manual pickup of HTGs that are related to carbon metabolism. These processes eventually produce a total of 4,554 HTG entries related to carbon metabolism, as listed in Supplementary Table S2 at https://doi.org/10.6084/m9.figshare.22154828.v1.

**Figure 1:**
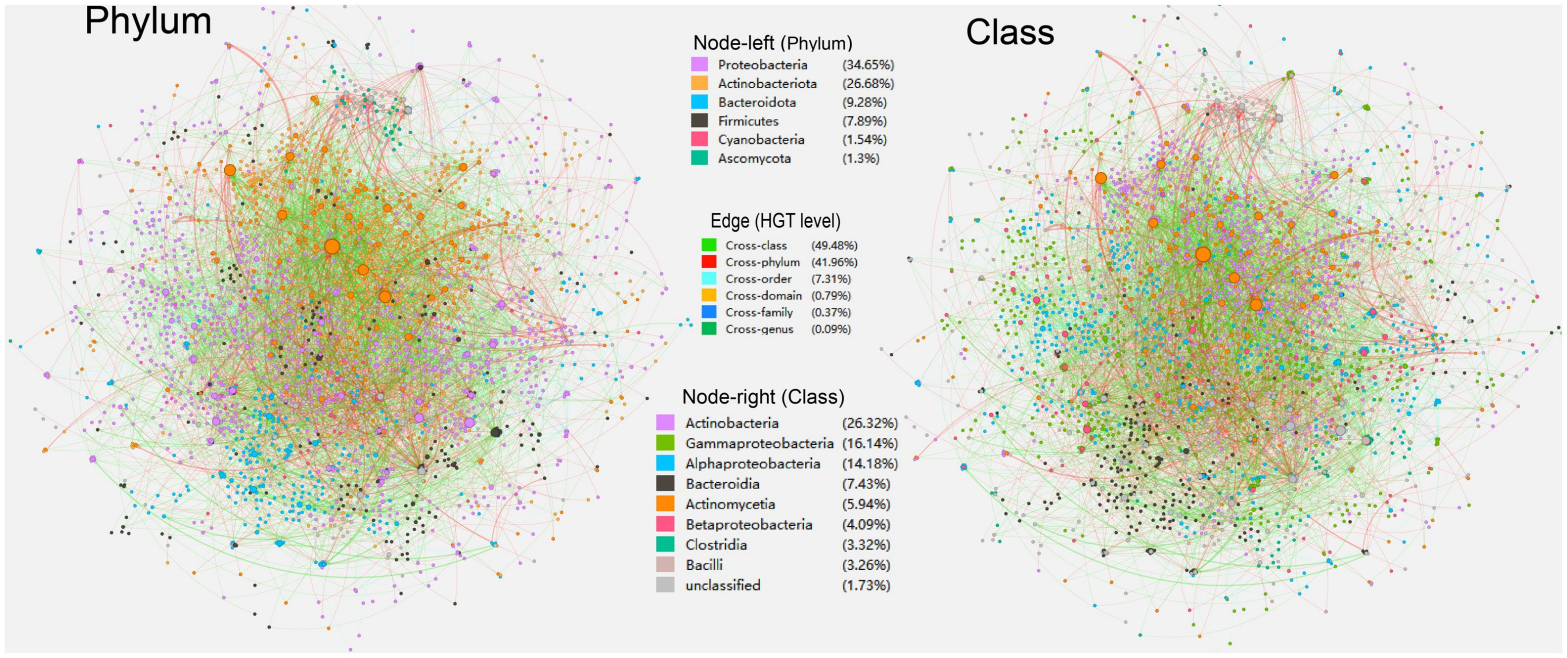
An overall directed horizontal gene transfer (HGT) network of carbon metabolic genes harbored by the soil-borne microbiota. Nodes represent genomes of species. The colors are marked according to the node’s taxonomy (left, phylum; right, class). The edges represent HGT events and are directed from the donor to the recipient.

The significance of HGT to biological diversification could be better understood through a network study of gene patterns amongst genomes. A directed phylogenomic network can be built using HGT inference techniques that consider the provider and receptor of the lateral gene delivery event (Popa et al., 2011). Similar to the methods used in previous work (Beiko et al., 2005), we established the phylogenomic HGT networks of carbon metabolic genes in soil-borne microbiota using HGT occurrences predicted to examine the HGT component within microbiological diversification (Figure 1). A baseline species (tested genomes) evolutionary tree’s exterior and internal nodes make up the HGT network’s vertex. Between the nodes, edges in the network refer to alleged gene transfer occurrences. If a gene relative is transferred across the nodes through a suspected HGT episode, these nodes are interconnected in the HGT networks. The HGT network’s edge weight is correlated with the amount of horizontally shared genes. Clusters are formed when specific subgroups of organisms have stronger connections among themselves than with other factions in the networks (Guimerà and Nunes Amaral, 2005; Gallos et al., 2007).

In the overall HGT network of carbon metabolic genes in soil-borne microbiota, a total of 6,770 nodes and 3,812 edges are present (Figure 1). Among the nodes, phyla Proteobacteria (34.65%), Actinobacteriota (26.68%), Bacteroidota (9.28%), and Firmicutes (7.89%) are predominant, followed by Cyanobacteria (1.54%) and Ascomycota (1.30%). Regarding specific classes, Actinobacteria (26.26%), Gammaproteobacteria (16.1%), Alphaproteobacteria (14.15%), Bacteroidia (7.42%), Actinomycetia (6%), Betaproteobacteria (4.08%), Clostridia (3.31%), and Bacili (3.25%) are dominant in the overall HGT network of carbon metabolic genes. The average path length (the average of the shortest route between any two nodes in the network) of the total HGT network is 1.55, indicating that the related carbon metabolic genes could be easily and rapidly transported through tested soil-borne microbiota. The HGT network’s thickest (weight > = 5) edges are mainly formed by cross-domain HGT events from archaeal phylum Haloarchaea to Bacteria (26/36 entries, 74.3%), with HGT from *Methanosarcina acetivorans* C2A (Haloarchaea) to S*treptomyces yangpuensis* fd2-tb (Actinobacteriota) having the highest HGT frequency (*n* = 14) (see Supplementary Table S3 at https://doi.org/10.6084/m9.figshare.22154828.v1). The chimeric nature and high externalization rate (especially on carbohydrate biosynthesis genes) of Haloarchaea have been highlighted previously (Galagan et al., 2002; Nelson-Sathi et al., 2012; Garushyants et al., 2015).

**Figure 2:**
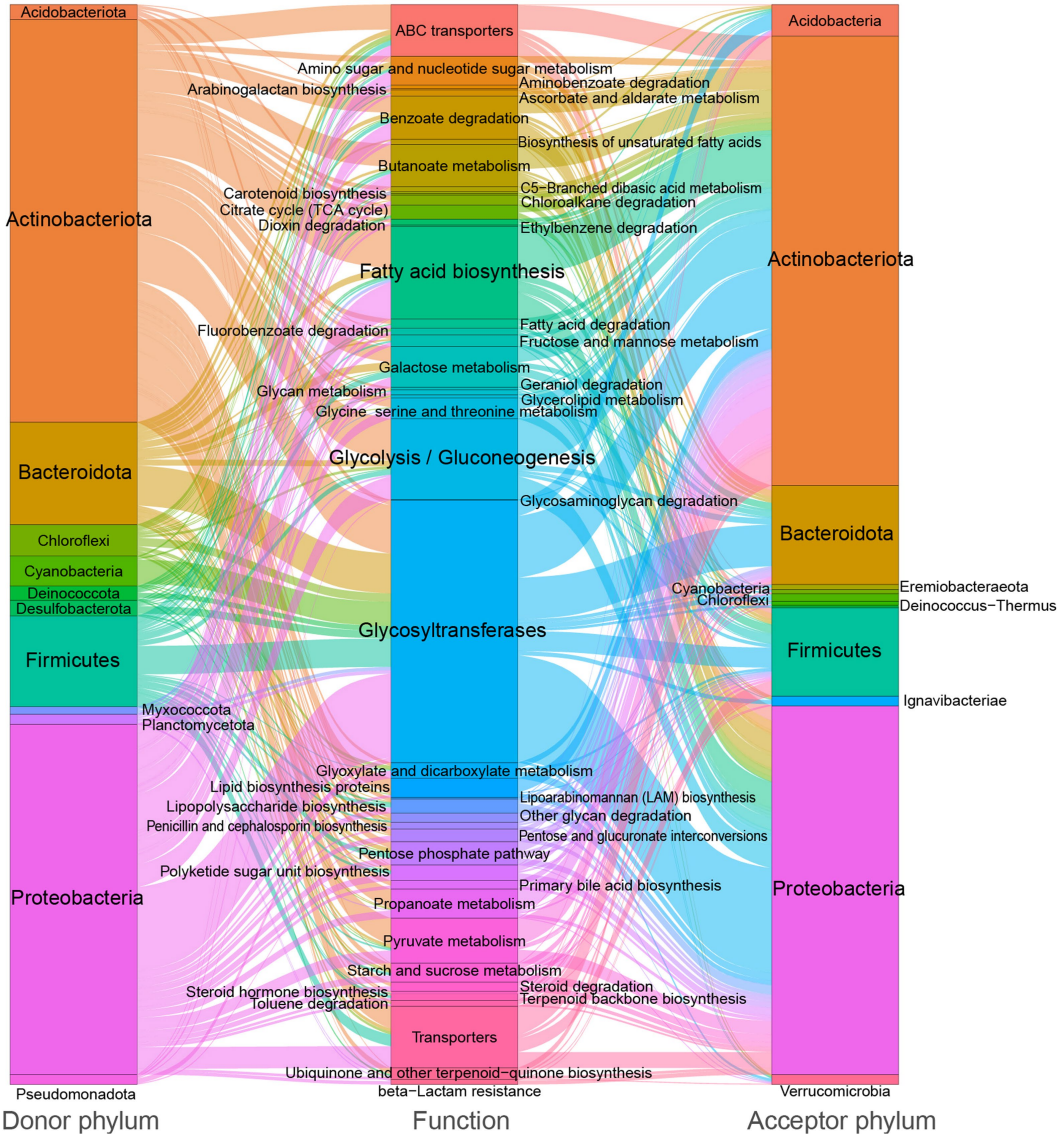
Sankey diagram showing the distribution of donor taxa, recipient taxa, and function categories of carbon metabolic genes within the soil-borne microbiota HGT network, with edge thickness and proportion indicating the relative frequency of transfer events.

In Kyoto Encyclopedia of Genes and Genomes (KEGG) functional assignments, glycosyltransferases (18.5%), glycolysis/gluconeogenesis (8.8%), carbohydrate-related transporters (7.9%), fatty acid biosynthesis (6.5%), benzoate degradation (3.1%), and butanoate metabolism (3.0%) are most frequently identified (Figure 2). These results suggest that soil-borne microbiota is involved in a range of carbon metabolic processes, particularly those related to the breakdown and transportation of carbohydrates and fatty acids. This may have important implications for soil health and ecosystem function.

We then used two classical network centralities, degree and betweenness, to examine what taxonomic groups are holding and shaping the HGT network (see Supplementary Tables S4, S5 at https://doi.org/10.6084/m9.figshare.22154828.v1). The most commonly used centrality measurement, degree, counts the edges that link a specific node. In a directed graph, the arrows are directional, pointing from one node (HGT donor) to another (HGT acceptor), so the number of arrows pointing to each node is its entry degree. In contrast, the number of arrows pointing away from this node is its outgoing degree. Of the top 30 nodes having the highest degree values (>25), class Actinomycetia occupies the most (43.2%) of these nodes (see Supplementary Table S4 at https://doi.org/10.6084/m9.figshare.22154828.v1), followed by classes Deltaproteobacteria (13.3%) and Betaproteobacteria (13.3%). In comparison, most other nodes (92.5%) in the overall HGT network of carbon metabolic genes in soil-borne microbiota display a degree value <5. In particular, the highest degree values in correspondent classes were seen in the genomes of *Kribbella soli* VKM Ac-2,540 (98, Actinomycetia), *Anaeromyxobacter* sp. Red232 (55, Deltaproteobacteria), *Paenibacillus piri* MS74 (41, Bacilli), *Pseudoduganella violaceinigra* DSM 15887(36, Betaproteobacteria), and *Pyrinomonas methylaliphatogenes* K22 (35, Blastocatellia).

Another centrality measurement, betweenness, measures how frequently a specific node is located on the shortest path possible between any pair of nodes within the network. Nodes with large betweenness centralities act as essential reservoirs or communicators for the common-good genetic codes. Of the top 30 nodes having the highest betweenness centrality values (>20) in the overall HGT network of carbon metabolic genes in soil-borne microbiota (see Supplementary Table S5 at https://doi.org/10.6084/m9.figshare.22154828.v1), class Actinomycetia also occupies most (60.0%) of these nodes, followed by class Deltaproteobacteria (10.0%). Primarily exhibiting the highest betweenness centrality values in correspondent classes are genomes of *Jiangella anatolica* GTF31 (558, Actinomycetia), *Pyrinomonas methylaliphatogenes* K22 (195.5, Blastocatellia), *Saccharicrinis fermentans* DSM 9555 (168, Bacteroidia), *Methylovulum miyakonense* HT12 (89, Gammaproteobacteria), and *Nitrosospira multiformis* ATCC 25196 (60.5, Betaproteobacteria). In comparison, most other nodes (98.2%) in the overall HGT network of carbon metabolic genes in soil-borne microbiota display a betweenness centrality value <5. On the other hand, nodes that display lower betweenness centralities either occupy peripheral positions in the network or form their linked constituents.

**Figure 3:**
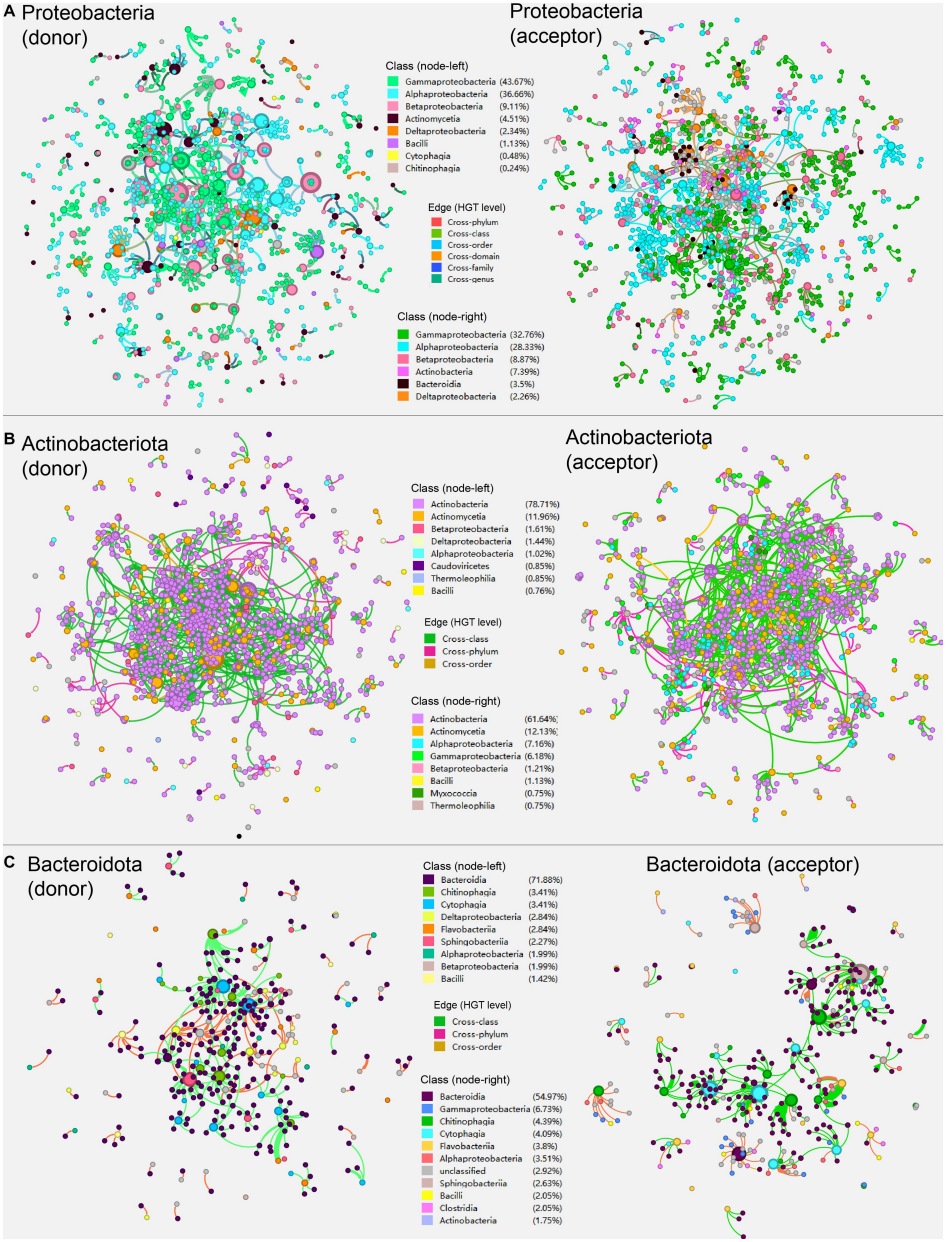
The sub-networks created by extracting corresponding taxonomic (e.g., Proteobacteria) nodes acting as HGT donors and acceptors, respectively, as well as extracting their direct edges according to the major phyla present in the overall HGT network: **(A)** Proteobacteria; **(B)** Actinobacteriota; **(C)** Bacteroidota. Nodes represent genomes of species. The edges represent HGT events and are directed from the donor to the recipient. The colors are marked according to the node’s taxonomy.

We further investigated the network’s community composition by segmenting it into sub-network according to the major phyla present in the overall HGT network (Figure 3). The sub-networks were created by extracting corresponding taxonomic (e.g., Proteobacteria) nodes acting as HGT donors and acceptors, respectively, as well as extracting their direct edges (Figure 3A). The donor and acceptor sub-networks of the same phyla generally exhibited similar network properties in comparison (Table 1). The same possesses for sub-network construction were applied to nodes of phyla Actinobacteria and Bacteroidota (Figures 3B,C).

**Table 1:** Network properties of the overall and sub-networks of carbon metabolic HGT detected in soil-borne microbiota.

HGT Network	Node number	Edge number	Average degree	Average weighted degree	average clustering coefficient	Diameter	Connected components	Modularity	Average path length	Average eigenvector centrality
Overall	6,770	3,812	1.126	1.343	0.001	5	143	0.863	1.55	0.136
Proteobacteria (donor)	1,241	1,173	0.945	1.041	0.001	3	123	0.922	1.106	0.08096
Proteobacteria (acceptor)	1,286	1,261	0.981	1.083	0.001	3	86	0.915	1.144	0.0653
Actinobacteria (donor)	1,179	1,325	1.124	1.38	0.001	5	63	0.828	1.73	0.052857
Actinobacteria (acceptor)	1,327	1,499	1.13	1.381	0.001	5	40	0.845	1.761	0.0604
Bacteroidota (donor)	353	346	0.98	1.159	0.0001	2	36	0.847	1.042	0.0261
Bacteroidota (acceptor)	342	330	0.965	1.108	0.0001	2	23	0.888	1.057	0.02255

In the sub-network that Proteobacteria members act as HGT donors (Figure 3A, left), Actinomycetia (4.51%) and Bacili (1.13%) are the most frequently observed classes outside the phyla Proteobacteria, indicating that classes Actinomycetia and Bacili are the major cross-phylum recipients of the genetic goods carried by Proteobacteria; whereas in the sub-network where Proteobacteria acting as HGT acceptor (Figure 3A, right), Actinobacteria (7.39%) and Bacteroidia (3.5%) are the most frequently observed classes outside the phyla Proteobacteria, suggesting that classes Actinobacteria and Bacteroidia are major cross-phylum donors of carbon metabolic genes to Proteobacteria.

Secondly, in the sub-network that Actinobacteriota members act as HGT donors (Figure 3B, left), Betaproteobacteria (1.61%), Deltaproteobacteria (1.44%), and Alphaproteobacteria (1.02%) the most frequently observed classes outside the phyla Actinobacteriota, indicating that these classes are the primary cross-phylum recipients of the genetic goods carried by Actinobacteriota; whereas in the sub-network where Actinobacteriota acting as HGT acceptor (Figure 3B, right), Alphaproteobacteria (7.16%), superior to Gammaproteobacteria (6.18%), and Betaproteobacteria (1.21%), stands for the most frequently observed classes outside the phyla Actinobacteriota, which indicates that classes from Proteobacteria like Alphaproteobacteria are major cross-phylum donors of carbon metabolic genes to Actinobacteriota.

Lastly, in the sub-network where Bacteroidota members act as HGT donors (Figure 3C, left), Deltaproteobacteria (2.84%), Betaproteobacteria (1.99%), and Alphaproteobacteria (1.99%) are the most frequently observed classes outside the phyla Bacteroidota, indicating that these classes are the major cross-phylum recipients of the genetic goods carried by Bacteroidota; whereas in the sub-network where Bacteroidota acting as HGT acceptor (Figure 3C, right), Gammaproteobacteria (6.73%), Alphaproteobacteria (3.51%), and Bacilli (2.5%) are the most frequently observed classes outside the phyla Bacteroidota, suggesting that these classes are major cross-phylum donors of carbon metabolic genes to Bacteroidota.

Network properties such as the node number, edge number, average (weighted) degree, as well as average path length were higher in the “acceptor” network than in the “donor” one in the corresponding networks of Proteobacteria and Actinobacteria, respectively. While the connector component (number of subgraphs in which each pair of nodes is connected) shows the opposite trend (higher in the “acceptor”). Average eigenvector centralities (that measure the transitive influence of nodes) decline from the Proteobacteria network to that of Actinobacteria, and, finally, Bacteroidota. Other network properties like modularity (measuring the structural strength and density of a network community) show only slight variations among groups (Table 1).

### 2.2. Carbon metabolic functions

**Figure 4:**
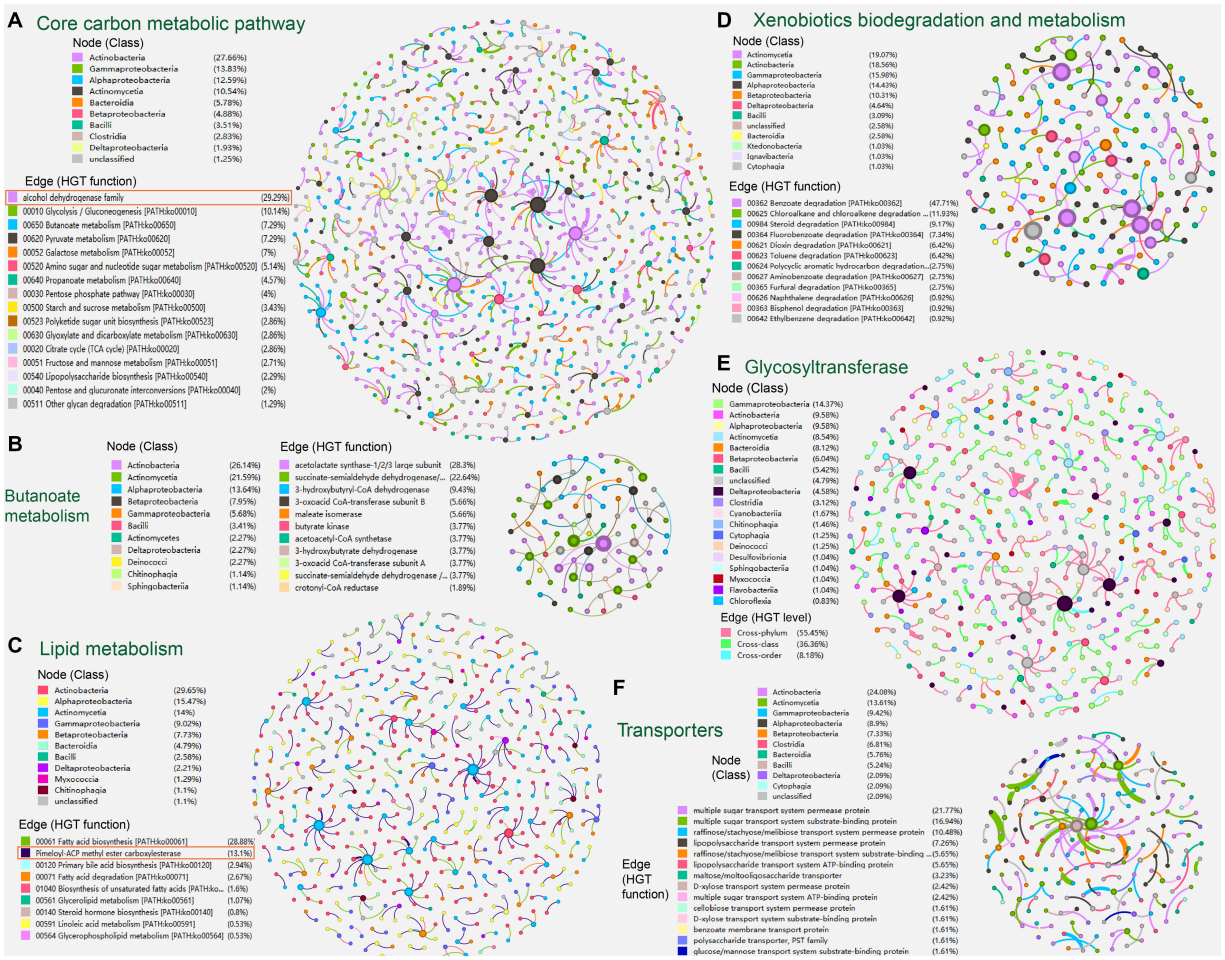
The sub-networks created by extracting HGT events (edges) corresponding to specific carbon metabolic functions: **(A)** core carbon metabolism; **(B)** butanoate metabolism; **(C)** lipid metabolism; **(D)** xenobiotics biodegradation and metabolism; **(E)** glycosyltransferase; **(F)** transport protein family. Nodes represent genomes of species. The edges represent HGT events and are directed from the donor to the recipient.

We further created sub-networks that extracted HGT events (edges) corresponding to specific carbon metabolic functions (e.g., core carbon metabolic pathway, fatty acid biosynthesis) and nodes acting as HGT donors and acceptors, respectively, as well as extracting their direct edges (Figure 4).

In the sub-network reflecting HGT episodes of core carbon metabolism (700 edges/genes, 882 nodes/genomes), genes attributed to pathways of glycolysis/gluconeogenesis (10.14%), butanoate metabolism (7.29%), pyruvate metabolism (7.29%), and galactose metabolism (7.00%) are predominant. Microbial classes Actinobacteria (27.66%), Gammaproteobacteria (13.83%), and Alphaproteobacteria (12.59%) are mostly seen (Figure 4A).

Alcohol dehydrogenase that metabolizes alcoholic substances (with 230 entries) is dominant in the current sub-network of core carbon metabolism (Figure 4A, marked with red rectangles) and ranks third in the list of most frequently identified carbon-metabolism-related horizontal transferred gene (HTG) from the soil-borne microbiota (see Supplementary Table S2 at https://doi.org/10.6084/m9.figshare.22154828.v1). Alcohol dehydrogenase exhibits a wide range of substrate specificity additionally, oxidizing mainly primary and secondary aliphatic alcohols when utilizingNAD^+^ as a co-substrate. It is also able to reduce aldehydes and ketones (Tsigos et al., 1998). Accordingly, HGT of alcohol dehydrogenase genes from prokaryotes and unicellular eukaryotes was also reported previously (Espinosa and Paz-Y-Miño-C, 2012; McCarthy and Fitzpatrick, 2016).

We further extracted the sub-networks of butanoate metabolism (53 edges, 88 nodes). In the sub-network, HGT genes encoding acetolactate synthase (28.3%), succinate-semialdehyde dehydrogenase (22.64%), and 3-hydroxybutyryl-CoA dehydrogenase (9.43%) are predominant. Microbial classes Actinobacteria (26.14%), Actinomycetia (21.59%), and Alphaproteobacteria (13.64%) are mostly seen (Figure 4B).

Likewise, in the sub-network reflecting HGT episodes of lipid metabolism (374 edges, 543 nodes), genes attributed to fatty acid biosynthesis (28.88%), primary bile acid biosynthesis (2.94%), and fatty acid degradation (2.67%) are predominant. Microbial classes Actinobacteria (29.65%), Alphaproteobacteria (15.47%), Actinomycetia (14.0%), Gammaproteobacteria (9.02%), and Betaproteobacteria (7.73%) are mostly seen at the same time (Figure 4C).

Unexpectedly, the pimeloyl-ACP methyl ester carboxylesterase (with 231 entries) is dominant in the current sub-network of lipid metabolism (Figure 4C, marked with red rectangles) and ranks second in the list of most frequently identified carbon-metabolism-related HTGs from the soil-borne microbiota (see Supplementary Table S2 at https://doi.org/10.6084/m9.figshare.22154828.v1). Pimeloyl-ACP methyl ester carboxylesterase catalyzes the hydrolysis of the ester bonds of pimeloyl-ACP esters that allow the synthesis of pimeloyl-ACP *via* the fatty acid synthetic pathway (Lin et al., 2010). Previous reports have also found pimeloyl-ACP methyl ester carboxylesterase located in MGEs, such as the plasmids of the psychrotolerant *Polaromonas* spp. isolated from Arctic and Antarctic glaciers (Ciok et al., 2018). It follows that 3-oxoacyl-ACP reductase (FabG) (with 74 entries) ranks third in the list of most frequently identified carbon-metabolism-related HTG from the soil-borne microbiota (see Supplementary Table S2 at https://doi.org/10.6084/m9.figshare.22154828.v1). FabG is the key enzyme directly responsible for the synthesis of 3-hydroxyacyl-ACPs in the fatty acid synthesis elongation cycle (Yu et al., 2019). As shown in previous studies, FabG has also been frequently observed in mobile genetic elements, like the plasmids of plant growth-promoting bacteria *Azospirillum brasilense* (Yu et al., 2019), and the plastid genomes of *Eustigmatophyte Algae* (Ševcíková et al., 2019).

Similarly, in the sub-network reflecting HGT episodes of xenobiotics biodegradation and metabolism (374 edges, 543 nodes), genes attributed to benzoate degradation (47.71%), chloroalkane/chloroalkene degradation (11.93%), steroid degradation (9.17%), fluorobenzoate degradation (7.34%), dioxin degradation (6.42%), and toluene degradation (6.42%) are predominant. At the same time, microbial classes Actinomycetia (19.07%), Actinobacteria (18.56%), Gammaproteobacteria (15.98%), Alphaproteobacteria (14.43%), and Betaproteobacteria (10.31%) are mostly observed (Figure 4D).

Regarding specific protein families, glycosyltransferase family (with 363 HGT entries) ranks first overwhelmingly in the list of most frequently identified carbon-metabolism-related HGT enzymes from the soil-borne microbiota (see Supplementary Table S2 at https://doi.org/10.6084/m9.figshare.22154828.v1). While microbial classes Gammaproteobacteria (14.37%), Actinobacteria (9.58%), Alphaproteobacteria (9.58%), Actinomycetia (8.54%), Bacteroidia, (8.12%), Betaproteobacteria (6.04%), and Bacilli (5.42%) are mostly observed to encode glycosyltransferase (Figure 4E). Glycosyltransferase is capable of catalyzing the sequential transfer of glycosyl moieties to the undecaprenyl phosphate carrier lipid during the early steps of polysaccharide synthesis, as well as various other acceptors like proteins, nucleic acid, and secondary metabolites (Garrison-Schilling et al., 2014; Zhang et al., 2020). Glycosyltransferase complexes are known to be involved in cell wall biosynthesis, protein glycosylation, and primary and secondary metabolism in both microbes and plants (Chang et al., 2003; Harholt et al., 2012), whose vital roles in enhancing metabolic flexibility and adaptive advantages might have endorsed the frequent HGT-driven expansions of glycosyltransferase across microbial species. Bacterial and viral glycosyltransferases determine their surface chemistry, influencing the interplay with their hosts (Brew et al., 2010). Our finding is also in line with previous investigations, which have found glycosyltransferase in Rickettsial plasmids (El et al., 2016). Also, the vascular wilt fungus *Verticillium* acquired glycosyltransferase involved in the synthesis of extracellular glucans by HGT from Proteobacteria (Lacroix and Citovsky, 2016). Besides, glycosyltransferase operons acquired *via* HGT were found to modify the O-antigen in *Salmonella enterica* serovar Typhi (Kintz et al., 2017).

Transport protein families (124 edges, 191 nodes) of organic carbon compounds were also visualized by sub-network creation (Figure 4F). In the sub-network, genes attributed to multiple sugar transport system (38.71%), raffinose/stachyose/melibiose transport system (16.13%), and lipopolysaccharide transport system (12.91%) are predominant. At the same time, microbial classes Actinobacteria (24.08%), Actinomycetia (13.61%), Gammaproteobacteria (9.42%), Alphaproteobacteria (8.90%), Betaproteobacteria (7.33%), and Clostridia (6.81%) are mostly observed to encode the transporters.

Also, there are other specific carbon metabolic enzymes enriched in the HGT network (see Supplementary Table S2 at https://doi.org/10.6084/m9.figshare.22154828.v1), such as beta-lactamase family enzymes (159 entries) that degrade multiple metabolites (e.g., antibiotics) for the use of the substrates as nutrients or carbon sources (Graham et al., 2016; Crofts et al., 2018; Maatouk et al., 2022), catechol 2,3-dioxygenase-like lactoylglutathione lyase (54 entries,COG0346) that could enable the degradation of phenol or lignin (Hupert-Kocurek et al., 2012), 2-polyprenyl-6-methoxyphenol hydroxylase (53 entries,COG0654) involved in the aerobic degradation of aromatic compounds (Trias et al., 2017) as well as quinol monooxygenase YgiN (34 entries) related to aerobic redox and physiology. Mapping these HTGs of carbon metabolism onto the correspondent KEGG pathways showed that most HTGs locate on the fringe of the metabolic cycles, and the core carbon metabolic sections rarely transfer (see Supplementary Figures S1–S5 at https://doi.org/10.6084/m9.figshare.22154828.v1).

## 3. Discussion

An editorial in the journal Science has advocated that more thorough investigations be conducted into the appearance and mechanics of HGT episodes (“Why does lateral transfer occur in so many species and how?”) (American Association for the Advancement of Science, 2005). We thoughtfully evaluated that the current study could have provided several suggestions for this subject.

A variety of soil-borne carbon-cycle HGT events of cross-class and even cross-phylum levels were discovered with considerably high transfer frequency (Figure 1). The constructed HGT networks demonstrate that genetic exchange across microbial genera is a significant contributor to microorganisms’ biodiversity and adaptivity. The characteristic of a “small-world system” found in previous HGT networks was also observed in our study, which refers to a network that has a small diameter in terms of the number of nodes and a handful of strongly linked nodes that allow for flux to flow across the system (Proulx et al., 2005). A small-world architecture in the HGT network indicates that significantly advantageous genes that arise in any microbe could transcend taxonomic boundaries and stretch another microbe through a limited amount of HGT episodes, where genomes with high betweenness can act as a link between otherwise unconnected parts of the network and transfer genes to numerous other genomes in the ecosystem with a small number of genetic transactions. Similarly, a recent study also found that the HGT rate was increased in organisms with similar ecological distributions (Zhou et al., 2021). Besides, the observed variations in network properties (Table 1) may have multiple implications for HGT events, highlighting the importance of understanding network dynamics in studying HGT events (Kunin et al., 2005; Kloesges et al., 2011; Popa et al., 2011; Li L. et al., 2020; Li X. et al., 2020). For example, a higher number of nodes and edges in the “acceptor” network may indicate a greater potential for gene transfer due to more opportunities for contact between bacteria. Similarly, higher degrees or connector components may create more interconnectedness amongst bacterial populations and facilitate the spread of genetic material. On the other hand, a longer average path length between nodes in a network may reduce the likelihood of gene transfer, as the distance between two bacteria would be higher and the chance of interaction would be lower. Moreover, differences in eigenvector centralities among network groups may illustrate the presence of influential nodes--nodes with high centrality values that may play significant roles in mediating gene transfer. Modularity can also impact the strength and density of community structures within a network, potentially creating barriers or pathways for gene transfer.

The phylum Proteobacteria was the most abundant taxon in soil samples, accounting for an average of 30% of metagenomic sequences (Guo et al., 2018). In keeping with this, our study found that nodes of classes, including Actinobacteria as well as Proteobacteria classes, such as Alphaproteobacteria and Gammaproteobacteria are extensively present in our overall HGT network of carbon metabolism (Figure 1), implying that HGT is widespread during the evolution of species in these classes. However, the sampling density of sequenced microbial genomes might be biased toward Proteobacteria, since the predominance of Proteobacteria in the genome dataset might be responsible for the enormous rate of HGTs within this group. Previous studies have confirmed that Proteobacterial MGEs constitute the major connected component in the virulence network, and extensive gene sharing exists among Actinobacteria and Gammaproteobacteria (Tamminen et al., 2012; Jiang et al., 2017). Previous studies have also validated experimentally the possibility of MGE-mediated HGT in soil samples, in which plasmids from the donor strains *Psudomonas putida* KT2440, *Escherichia coli* MG1655, and *Kluyvera* sp. can be transferred to a wide range of bacterial phyla from agricultural soils (Klümper et al., 2015), including (α–ε) Proteobacteria, Acidobacteria. Actinobacteria, Bacteroidetes, Firmicutes, Fusobacter, Gemmatimonadetes, Planctomyces, spirochaetes, Candidate division TM7, Verrucomicrobia as well as Eukaryote (Zhang et al., 2015).

Externalized genes carried by MGEs could act as containers for the shared genetic pool (Norman et al., 2009). In our study, uneven functional distributions of the externalized gene are also discovered, in which HTGs are mostly incorporated into the peripheral functions of the carbon metabolic pathway (e.g., nutrient transport and dispensable reaction). In contrast, the core metabolic sections (e.g., intermediate reactions and biomass production) of putative competent significance are mostly evolutionarily unvaried (see Supplementary Figures S1–S5 at https://doi.org/10.6084/m9.figshare.22154828.v1). Similar findings were also reported previously. For example, according to research on the horizontally gained genes within the *E. coli* metabolic pathways (Pál et al., 2005), HGT is more common among proteins engaged in the absorption and consumption of resources than those responsible for the generation of biomass, which suggested that their function influences the HGT probability of metabolic genes in the internal metabolic pathway. This uneven functional distribution could also be explained by the “complexity hypothesis” (Jain et al., 1999; Muller et al., 2018), which proposed that proteins in a complicated system, like ribosomal systems or core metabolic cycles, are specialized to work. Decreased adaptation of the microbial recipients will arise from an HGT incidence that leads to substituting such a gene with a less-suited counterpart. The proportional influence of functionality type and the number of interactive participants on HGT incidence was also investigated, demonstrating that the “complexity theory” still holds up in the genome-scale analyses (Cohen et al., 2011).

On the other hand, soil multi-functionality is affected by the environment and by microbial community composition and diversity (Zheng et al., 2019). Environmental factors in the soil also impact the HGT processes and lead to uneven distribution. The microbial HGT rate in soil relies on environmental stress variables like surface temperatures, pH (Rochelle et al., 1989), soil composition (Richaume et al., 1992), and wetness (Richaume et al., 1989). The gene-sharing network revealed strong correlations between gene connectivity and the trailed soil variables (Zhou et al., 2010). Besides, the persistence and transportation of genetically modified bacteria were impacted by fluidity in subsoil (Trevors et al., 1990). In terms of biological variables, the existence of fungi (Sengeløv et al., 2000), protozoa (Johannes Sørensen et al., 1999), and roundworms (Daane et al., 1996, 1997) could also impact sexual plasmid transmission in soil. Moreover, the generation of leachate and root elongation looked to be the main contributors to the incidence of HGT inside the rhizosphere (Mølbak et al., 2007). Transmission rates were roughly 10 times lower in the bean and cereal rhizospheres than in the control soils (Musovic et al., 2006). Lastly, the other key factor contributing to the rise in local MGE quantities and HGT incidence in this environment is the implementation of manure, pesticides, and antimicrobials to the land (Gotz and Smalla, 1997). Correspondently, various organic degradative genes were found mobilized among soil-borne microbiota *via* HGT in our study (Figure 4D), whose catabolic activities might be further applied for bio-remediation of polluted environments (Top et al., 2002; Nojiri et al., 2004). It is recently confirmed that genes of microalgal origin have conferred *Caenorhabditis elegans* the ability to degrade cyanogenic toxins (Wang et al., 2022). Previous studies also found that functional categories “biosynthesis and degradation of surface polysaccharides and lipopolysaccharides” and “DNA regulation and modification” tend to be enriched in the HGT entries. In consistence, we found glycosyltransferase in our study as the most abundant protein encoded by HTG related to carbon metabolism. The glycosyltransferase family was reported to be involved in the glycosylation and modifications of biomolecules, including the bases in DNA, which might alter the host’s gene expression pattern (Iyer et al., 2013). Also, it was reported that glycosyltransferases were significantly enriched in horizontally transferred genes in the human gut, while soil microbiota has similar expansions of the glycosyltransferase repertoires as the gut (Lozupone et al., 2008). Another generally enriched functional category in the HGT profile is metabolite transporter (Cordero and Hogeweg, 2009; Paquola et al., 2018), which is also reflected by our results (Figure 4F).

In conclusion, the inter-microbe HGT genetic traits in soil-borne microbiota genetic sequences that we recognized through our assessments, as well as their involvement in carbon metabolism and resilience to various environmental stressors typically found in territory ecosystems, suggest a pervasive and substantial effect of HGT on the evolvement of microbes. Nevertheless, the information we have provided here is not thorough. The examples of inter-microbe HGT documented so far represent just the tip of a giant biological iceberg. Upcoming studies are expected to offer a more intriguing view of both the scope and the biological importance of HGT in microbes as increasing numbers of genomic data of higher quality are becoming accessible for yet more branches in the tree of life for microbes. Further work will be necessary to evaluate the occurrence of HGT in the vast, heterogeneous, and isolated environment of the terrestrial subsurface and to assess the full impact of gene transfer on terrestrial subsurface microbial evolution.

## 4. Materials and methods

We first queried and retrieved all the items in the public database (Genbank/IMG) with the keyword “soil” within the “biosample” or “habitat” regions. We have then chosen and downloaded from the public database (Genbank/IMG) the resultant high-quality 764 genomes of soil microbial isolates as listed in Supplementary Table S1 at https://doi.org/10.6084/m9.figshare.22154828.v1 for downstream analyses. The Integrated Microbial Genomes Annotation Pipeline (IMGAP) v.5.0 (Markowitz et al., 2010) under default mode was used to identify horizontally transferred genes (HTGs) in the genetic sequences of individual collected soil-borne microbiota, as conducted in previous studies (Li L. et al., 2020; Li X. et al., 2020; Li et al., 2021, 2022). It used the following criteria to determine which genes inside the trialed genomes were horizontally transferred from remote descendants: genes with the finest BLASTP matches (most significant bit scores) or over 90% of the best hits discovered beyond the phylogenetic clade of the trialed genome (i.e., from remote phylum, class, etc.) and with lower-scoring matches or no hits within the original phylogenetic clade of the trialed genome. The HTG sequences were annotated with the eggnog mapper v.2.0 (Huerta-Cepas et al., 2019). This resulted in a total of 37,481 HTG entries, followed by the pickup of HTGs that are related to carbon metabolism according to the Kyoto Encyclopedia of Genes and Genomes (KEGG) classification. These processes eventually produce a total of 4,554 HTG entries related to carbon metabolism. We then used the Gephi network visualization package^1^ for network visualization and exploration of carbon metabolism HTGs. Fruchterman Reingold and Openord layout approaches were used for network visualization in the Gephi. The graphical user interface of the KEGG pathway map color tool was applied for coloring map objects, including split-coloring and gradation^2^.

## Data availability statement

The original contributions presented in the study are included in the article/supplementary material, further inquiries can be directed to the corresponding authors.

## Author contributions

LL and ZL conceived and designed the research. YL, QX, and ZX provided the computing sources. LL, DM, ZY, and HY analyzed the data. LL wrote the manuscript. All authors contributed to the article and approved the submitted version.

## Funding

This research was supported by the key project of Science and Technology of Hunan Branch of China National Tobacco Corporation (CD2022KJ01, 2022431021240240, HN2021KJ05), Natural Science Foundation of Changsha (No. kq2202089), Fundamental Research Funds for the Central Universities of Central South University (No. 2022ZZTS0420), Hunan International Scientific and Technological Cooperation Base of Environmental Microbiome and Application (No. 2018WK4019), and key project of Science and Technology of China National Tobacco Corporation (No. 110202201004) (JY-04).

## Conflict of interest

YL, QX, and ZX were employed by Zhangjiajie Tobacco Company of Hunan Province.

The remaining authors declare that the research was conducted in the absence of any commercial or financial relationships that could be construed as a potential conflict of interest.

## Publisher’s note

All claims expressed in this article are solely those of the authors and do not necessarily represent those of their affiliated organizations, or those of the publisher, the editors and the reviewers. Any product that may be evaluated in this article, or claim that may be made by its manufacturer, is not guaranteed or endorsed by the publisher.

## Footnotes

^1^https://gephi.org

^2^https://www.kegg.jp/kegg/mapper/color.html
